# Physical Activity and Untreated Dental Caries in Brazil: Implications for Population-Based Caries Risk Assessment

**DOI:** 10.3390/dj14080514

**Published:** 2026-08-12

**Authors:** Maria Clara dos Reis, Pedro Paulo Menezes Scariot, Leonardo Henrique Dalcheco Messias

**Affiliations:** Research Group on Technology Applied to Exercise Physiology—GTAFE, Health Sciences Postgraduate Program, São Francisco University, Bragança Paulista 12916-900, São Paulo, Brazil; maria.clara.reis@mail.usf.edu.br (M.C.d.R.); pedro.scariot@usf.edu.br (P.P.M.S.)

**Keywords:** physical activity, untreated dental caries, oral health, dental caries, oral health indicators

## Abstract

**Background/Objectives:** Oral diseases are a major public health challenge and share behavioral and socioeconomic determinants with other noncommunicable diseases. Although physical activity has been proposed as one of these shared determinants, evidence at the population level remains limited. This study investigated the association between physical activity and oral health indicators using nationwide Brazilian surveillance data. **Methods:** An ecological study was conducted using aggregated data from the Surveillance System for Risk and Protective Factors for Chronic Diseases by Telephone Survey (VIGITEL) and the Brazilian National Oral Health Survey (SB Brasil) (2022–2023). Physical activity participation and weekly volume were evaluated against untreated dental caries, toothache, pain intensity, tooth loss, and the Decayed, Missing, and Filled Teeth (DMFT) index. Weighted multivariable linear regression models with heteroskedasticity-consistent standard errors were applied. **Results:** The analysis included 28,707 VIGITEL participants and 14,753 SB Brasil observations. After adjustment, physical activity participation was inversely associated with untreated dental caries, whereas weekly physical activity volume showed no association with any oral health outcome. No significant associations were observed for tooth loss, dental pain, pain intensity, or the DMFT index. **Conclusions:** Higher population-level participation in physical activity was associated with a lower prevalence of untreated dental caries but not with other oral health indicators. These findings may suggest that physical activity and oral health share common behavioral and social determinants, supporting integrated public health strategies targeting upstream risk factors.

## 1. Introduction

Oral diseases remain a major public health challenge worldwide, affecting an estimated 3.69 billion people in 2021 [1,2]. Untreated dental caries, periodontal disease, and tooth loss account for most of this burden, with untreated caries in permanent teeth remaining the single most prevalent health condition globally, affecting approximately 2.24 billion individuals. In Brazil, the burden of oral diseases has also remained substantial. The most recent National Oral Health Survey [3] reported that approximately 44% of adults had at least one untreated carious tooth, a prevalence virtually unchanged since 2010 despite advances in oral health care [4]. Although important improvements have been observed in tooth retention over recent decades, marked socioeconomic inequalities in oral health outcomes persist, disproportionately affecting socially disadvantaged populations [5,6].

The persistence of oral diseases despite advances in dental care highlights that their determinants extend well beyond clinical factors. Oral diseases are now recognized as part of the broader noncommunicable disease framework because they share common behavioral, biological, and social determinants with conditions such as cardiovascular disease, diabetes, obesity, and several cancers [5,7,8]. This concept is formalized by the Common Risk Factor Approach [9], which proposes that population-wide interventions targeting shared modifiable risk factors, including excessive sugar consumption, unhealthy diet, tobacco and alcohol use, and physical inactivity, can simultaneously reduce the burden of oral and systemic diseases. Reflecting this growing recognition, recent authors have advocated expanding the World Health Organization’s (WHO) 5 × 5 noncommunicable disease framework, which addresses five major disease groups (cardiovascular diseases, cancer, chronic respiratory diseases, diabetes, and mental health conditions) and five shared modifiable risk factors (tobacco use, harmful alcohol use, unhealthy diet, physical inactivity, and air pollution), to a 6 × 6 model by formally incorporating oral diseases and free sugars into the global noncommunicable disease (NCD) agenda [10].

Evidence supporting the hypothesis that physical activity and oral health share common behavioral and socioeconomic determinants has grown in recent years but remains relatively limited. Population-based studies consistently suggest that physically active individuals experience better oral health outcomes than their inactive counterparts. In a nationwide study including 17,777 Spanish adults, sufficient physical activity was associated with a 28% lower likelihood of dental caries [11]. Similarly, data from Finland demonstrated that both low physical activity and poor cardiorespiratory fitness were independently associated with a higher prevalence of dentine caries [12]. Beyond dental caries, leisure-time physical activity has also been associated with lower odds of periodontitis, whereas prolonged sedentary behavior has emerged as an independent risk factor for periodontal disease across different populations [13,14,15].

Although the biological mechanisms remain incompletely understood, current evidence suggests that these associations may reflect both behavioral and physiological pathways. Physically active individuals are more likely to adopt healthier lifestyles, including better dietary and oral hygiene habits, while regular physical activity also reduces systemic inflammation and improves metabolic regulation, mechanisms that may contribute to maintaining oral health [13,16]. Nevertheless, most available evidence is derived from cross-sectional studies, limiting causal inference. This knowledge gap is particularly relevant in Brazil, where substantial regional and socioeconomic inequalities in oral health persist despite advances in public dental care policies [6,17]. Disadvantaged populations continue to experience a disproportionately higher burden of oral diseases and reduced access to preventive dental care [18,19]. These inequalities are accompanied by the clustering of health-related behaviors, such as physical activity, dietary habits, sedentary behavior, and oral hygiene practices, which tend to coexist within the same individuals and social groups rather than occurring in isolation [20,21,22]. Such clustering reinforces the rationale of the Common Risk Factor Approach and suggests that population-level patterns of physical activity may also be reflected in oral health outcomes.

Brazil offers a unique opportunity to investigate this hypothesis through two nationwide surveillance systems. The Surveillance System for Risk and Protective Factors for Chronic Diseases by Telephone Survey (VIGITEL) [23] routinely monitors physical activity and other behavioral risk factors, whereas SB Brasil provides comprehensive epidemiological data on oral health. To our knowledge, these databases have not previously been integrated to examine ecological associations between physical activity and oral health indicators. Linking these complementary surveillance systems allows the evaluation of whether associations consistently observed at the individual level are also detectable at the population level, generating evidence that may support integrated health promotion strategies targeting shared behavioral determinants. Therefore, the present study investigated the association between physical activity indicators and oral health outcomes using an ecological analysis of Brazilian national surveillance data. Analyses were stratified by macro-region, sex, and age group. We hypothesized that populations with a higher prevalence of physical activity would present more favorable oral health indicators, consistent with the clustering of health-promoting behaviors proposed by the Common Risk Factor Approach, even after adjustment for demographic and regional characteristics.

## 2. Methods

### 2.1. Design

This study employed an ecological analytical design using aggregated data from Brazilian population-based surveillance systems for the years 2022 and 2023. The analysis was conducted using ecological strata defined by the combination of Brazilian state capital, sex, and age group. Macro-region was included as an adjustment variable in subsequent regression models. Analyses were conducted in line with the Reporting of Studies Conducted using Observational Routinely Collected Health Data (RECORD) (Supplementary File S1) guidelines [24]. The study relied on publicly available, anonymized secondary data, complying with Brazilian regulations for the use of health surveillance information.

### 2.2. Data Source

Data were obtained from two national surveillance systems. Indicators of physical activity were derived from the VIGITEL, which annually monitors health behaviors among adults living in Brazilian state capitals and the Federal District. Data on oral health outcomes were obtained from the National Oral Health Survey (SB Brasil). Clinical oral examinations were performed by previously trained and calibrated dentists following the standardized diagnostic criteria recommended by the WHO and the methodological protocol adopted by SB Brasil. Examiner calibration was conducted using a reference standard, and only examiners achieving a minimum inter-examiner agreement of Kappa ≥ 0.61 were certified for field data collection, as detailed in the official SB Brasil methodological report [25]. All clinical oral health outcomes analyzed in this study, including untreated dental caries, tooth loss, and DMFT, were derived from the standardized SB Brasil examination protocol. Dental caries was assessed using the WHO Oral Health Surveys: Basic Methods criteria adopted by SB Brasil rather than the International Caries Detection and Assessment System (ICDAS-II). Consequently, only cavitated carious lesions were recorded, and non-cavitated (incipient) lesions were not included in the assessment. Toothache was obtained through participant self-report using the standardized SB Brasil questionnaire, as pulp vitality testing is not part of the survey protocol.

The selected time frame was determined by the availability of oral health data, as the most recent complete report covered the years 2022 and 2023. To ensure geographic comparability with VIGITEL, only participants residing in the 27 Brazilian state capitals were included from the SB Brasil database. Consequently, both datasets represented the same geographic sampling frame before aggregation by capital, sex, and age group. Therefore, the two databases were matched using identical geographic (state capital), sex, and age-group strata, ensuring that exposure and outcome estimates referred to the same target populations in the ecological analyses.

### 2.3. Variables

Physical activity data were obtained from VIGITEL and comprised two exposure variables. Physical activity participation was defined as the proportion of adults classified as engaging in leisure-time physical activity according to the standardized VIGITEL methodology, which combines the reported type, frequency, and duration of leisure-time physical activity. Weekly physical activity volume was expressed as the mean duration of leisure-time physical activity (minutes/week), calculated by VIGITEL from the reported frequency and duration of activity. Because these measures are based on self-reported telephone interviews, they are subject to recall and social-desirability bias. Oral health outcomes were obtained from the SB Brasil 2023 and included the prevalence of untreated dental caries (%), prevalence of self-reported toothache (%), mean tooth pain intensity (0–10 numeric rating scale), mean number of missing teeth, and the mean Decayed, Missing, and Filled Teeth (DMFT) index. All variables were aggregated according to Brazilian state capital, sex, and age group, yielding 216 ecological strata (27 capitals × 2 sex categories × 4 age groups), with each stratum constituting a single observational unit for the ecological analyses. Macro-region was retained as an adjustment variable in the regression models. A detailed description of all study variables, their definitions, units of measurement, and data sources is provided in Table 1.

### 2.4. Data Handling and Analytical Considerations

Data from the different sources were harmonized prior to analysis to ensure consistency across variables and time points. Because the datasets originated from distinct surveillance systems, harmonization was performed using identical stratification variables (Brazilian state capital, sex, and age group). Age categories were harmonized as 18–29, 30–39, 40–49, and ≥60 years. Adults aged 50–59 years were excluded because no directly comparable age category was available in the SB Brasil survey, thereby ensuring complete consistency between the two national databases. For analytical purposes, variables were expressed as population-level estimates, including prevalences (for binary outcomes) and means (for continuous measures). Given the ecological nature of the study, the unit of analysis was the ecological stratum defined by the combination of Brazilian state capital, sex, and age group rather than the individual. As such, interpretations were restricted to population-level associations, avoiding inferences at the individual level. To account for differences in sample size across strata, analyses were weighted using the number of individuals contributing to each estimate, improving the representativeness of the results.

### 2.5. Statistical Methods

All analyses were conducted using the R statistical software 4.5.2 (R Foundation for Statistical Computing, Vienna, Austria). Data processing and organization were performed using the dplyr package (version 1.2.1), while graphical representations were generated using ggplot2 (version 4.0.3). Descriptive analyses were initially performed to explore the distribution of physical activity and oral health indicators across regions and population strata. These patterns were visualized using scatter plots and smoothed trends to facilitate interpretation. To investigate the association between physical activity and oral health outcomes, separate weighted multivariable linear regression models were fitted using 216 ecological strata. Each oral health indicator was analyzed as a dependent variable in a separate model, with physical activity indicators included as the main independent variables. All models were adjusted for sex, age group, and macro-region, allowing the estimation of associations independent of these structural factors. Regression coefficients (β) and corresponding 95% confidence intervals were reported. To improve the robustness of the estimates, heteroskedasticity-consistent HC3 standard errors were applied. Statistical significance was defined as *p* < 0.05.

## 3. Results

**Figure 1:**
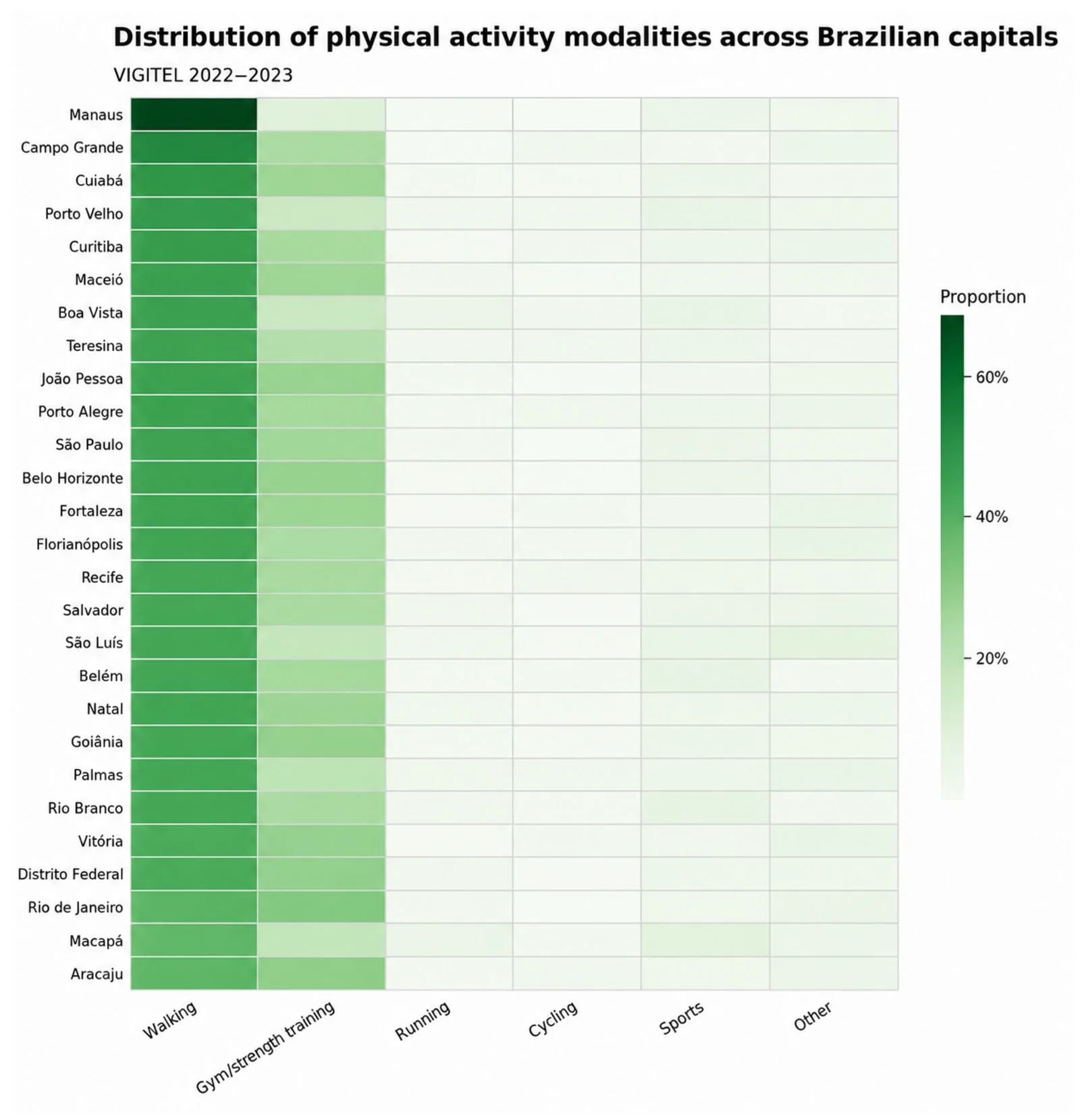
Distribution of physical activity modalities across Brazilian capitals based on the Surveillance System for Risk and Protective Factors for Chronic Diseases by Telephone Survey (VIGITEL) data (2022–2023). The heatmap displays the proportion of each modality within capitals, with green color intensity indicating higher relative prevalence. Modalities include walking, gym/strength training, running, cycling, sports, and other activities.

The final analytical dataset comprised 216 ecological strata derived from the combination of 27 Brazilian state capitals, two sex categories, and four age groups. These strata were generated from information obtained from 28,707 individuals surveyed through the VIGITEL system and 14,753 observations from the SB Brasil program. The distribution of oral health indicators and physical activity across Brazilian capitals is presented in Supplementary File S2. The distribution of physical activity modalities across Brazilian capitals is presented in Figure 1. Across capitals, walking consistently represented the most prevalent modality, followed by gym/strength training, while running, cycling, and sports showed comparatively lower proportions. The “other” category also accounted for a substantial share in several capitals.

**Figure 2:**
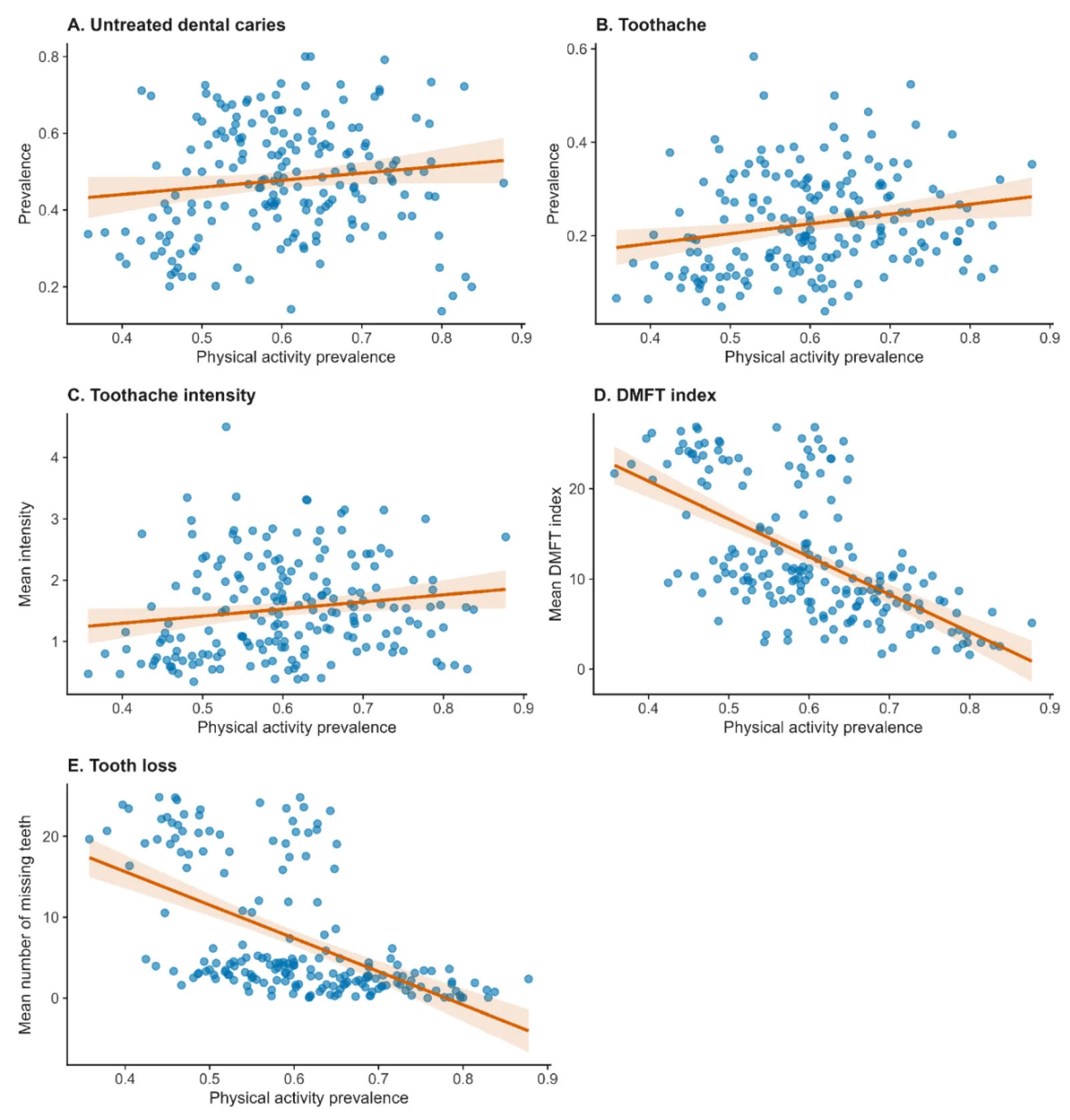
Crude relationships between physical activity prevalence and oral health outcomes across ecological strata. Scatterplots represent untreated dental caries (**A**), toothache prevalence (**B**), mean toothache intensity (**C**), mean Decayed, Missing, and Filled Teeth index (**D**), and mean tooth loss (**E**). Solid lines indicate fitted linear trends with corresponding 95% confidence bands.

Crude relationships between physical activity prevalence and oral health outcomes are presented in Figure 2. Visual inspection of the fitted linear trends suggests a slight positive pattern between physical activity and untreated dental caries (Figure 2A), toothache prevalence (Figure 2B), and mean toothache intensity (Figure 2C), although these relationships appear weak and highly dispersed. In contrast, more pronounced inverse patterns were observed for cumulative oral health indicators. Both the mean DMFT index (Figure 2D) and mean tooth loss (Figure 2E) demonstrated clear negative trends, indicating lower values in strata with higher physical activity prevalence. However, given that these analyses are based on unadjusted scatterplots, no formal statistical inference should be drawn from these patterns.

**Figure 3:**
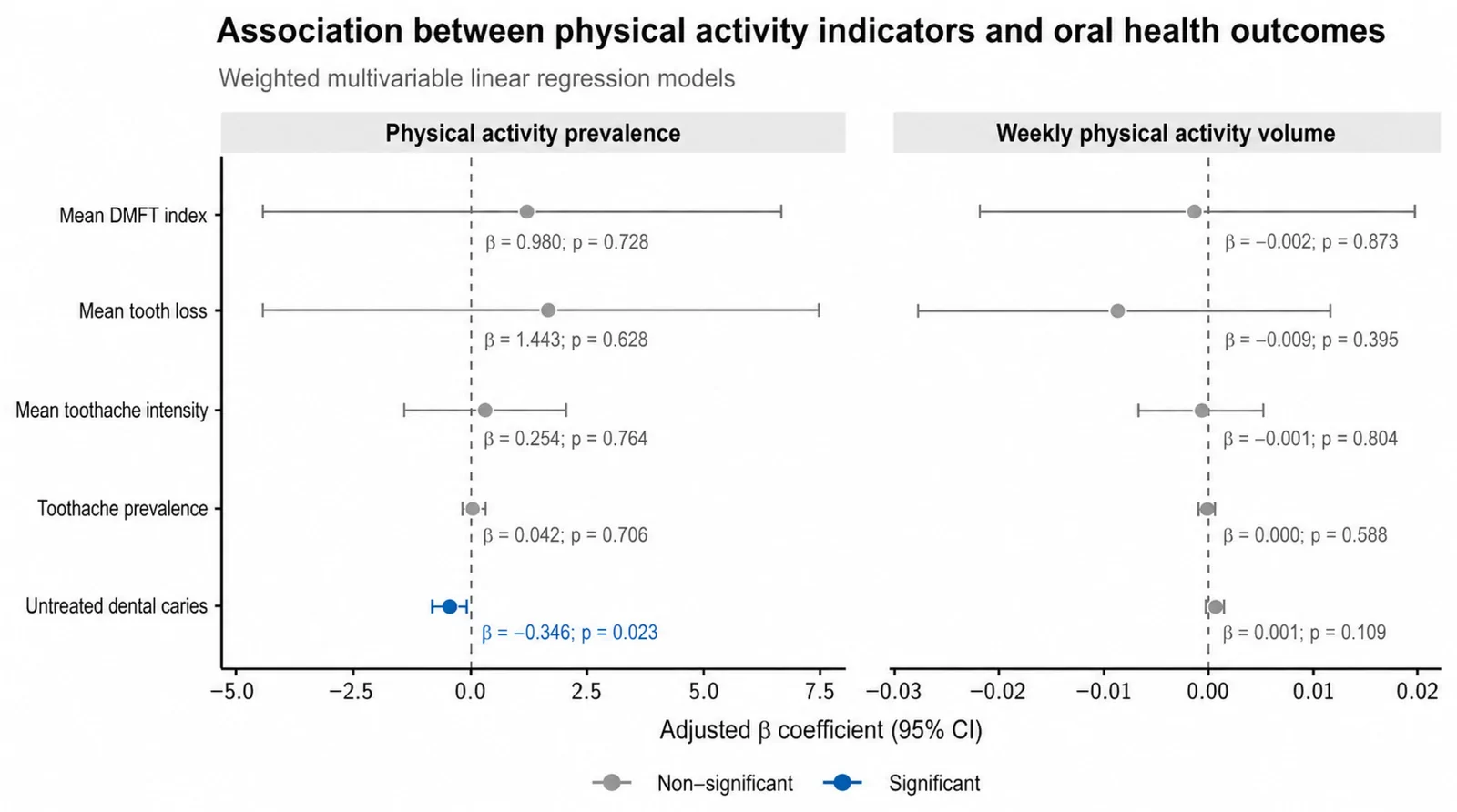
Adjusted associations between physical activity indicators and oral health outcomes based on weighted multivariable linear regression models. Results are presented as β coefficients with 95% confidence intervals for physical activity prevalence and weekly physical activity volume across all outcomes.

Adjusted associations between physical activity indicators and oral health outcomes are presented in Figure 3. Overall, no consistent associations were observed between physical activity and most oral health outcomes after multivariable adjustment. Both physical activity prevalence and weekly volume showed null relationships with toothache prevalence, toothache intensity, mean tooth loss, and mean DMFT index. Notably, a statistically significant inverse association was identified only for untreated dental caries in relation to physical activity prevalence. However, this pattern was not replicated when physical activity was operationalized as weekly volume. Complete results from the weighted multivariable linear regression model are presented in Supplementary File S3. For untreated dental caries, apart from the physical activity prevalence association, significant effects of sex, multiple age groups, and the North region were also obtained. In the toothache model, only the oldest age group and the North region showed significant negative associations. Similarly, toothache intensity was significantly lower in the oldest age group and in the North region. For DMFT, strong positive associations were observed across all older age groups, while the South region showed a significant negative association. A comparable pattern was identified for tooth loss, with significant increases across advancing age groups and a significant reduction observed in the South region.

**Figure 4:**
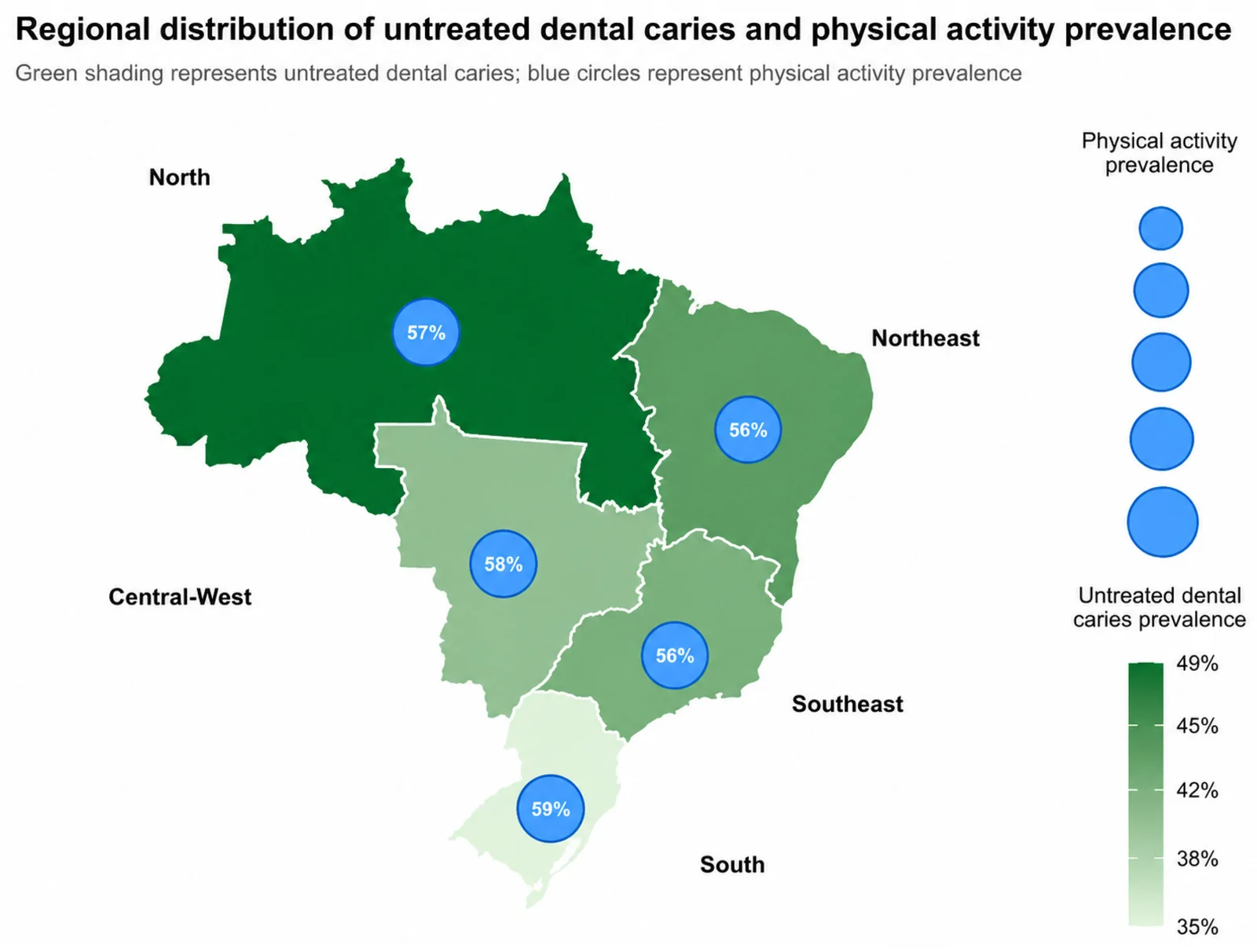
Regional distribution of untreated dental caries and physical activity prevalence across Brazilian macro-regions. Green shading represents untreated dental caries prevalence, while blue circles indicate physical activity prevalence, with circle size proportional to the magnitude of the estimate.

The regional distribution of untreated dental caries and physical activity prevalence is presented in Figure 4. Marked variability was observed across Brazilian macro-regions for both indicators. Untreated dental caries prevalence ranged from lower values in the South to higher values in the North, while physical activity prevalence showed variation across all regions, with the highest levels observed in the South and the lowest in the Southeast.

## 4. Discussion

At the population level, this ecological analysis indicates that Brazilian regions and demographic strata with a higher prevalence of physical activity engagement also present a lower prevalence of untreated dental caries, an association that remained significant after adjustment for sex, age group, and macro-region. This pattern did not extend, however, to toothache, pain intensity, tooth loss, or the DMFT index, nor was it reproduced when physical activity was expressed as weekly volume rather than participation. Because the unit of observation in this study is the population stratum rather than the individual, these findings are best interpreted as evidence of population-level covariation between physical activity and untreated caries prevalence, rather than as evidence of an individual causal effect of exercise on caries risk. Given the multiplicity of exposure–outcome tests performed, however, this nominally significant association should be regarded as hypothesis-generating rather than confirmatory evidence and interpreted with appropriate caution.

This population-level pattern is broadly consistent with findings reported in recent individual-level studies across different populations. In a nationwide study of 17,777 Spanish adults, sufficient physical activity was associated with a 28% lower likelihood of dental caries after adjustment for sociodemographic and behavioral factors [11]. Similarly, the Finland Cohort demonstrated that both low physical activity and poor cardiorespiratory fitness were independently associated with higher dentine caries prevalence [12], while comparable inverse associations have recently been observed among Chinese university students [26]. The convergence between these individual-level observations and the present ecological findings is consistent with the Common Risk Factor Approach, which recognizes physical inactivity as one of several modifiable behaviors that cluster with unhealthy diet, poor oral hygiene, tobacco use, and socioeconomic disadvantage to shape the population distribution of oral diseases and other noncommunicable conditions [7,9,27].

A plausible explanation for the observed population-level association is the clustering of health-promoting behaviors rather than a direct protective effect of physical activity on dental caries. Health behaviors rarely occur in isolation. Instead, physical activity, healthy dietary patterns, and appropriate oral hygiene practices tend to coexist within the same individuals and population groups, whereas physical inactivity commonly clusters with an unhealthy diet, sedentary behavior, and poor oral hygiene [20]. Similar patterns have been documented in Brazil, where oral hygiene practices, dietary habits, and physical activity are embedded within broader behavioral profiles shaped by shared socioeconomic and environmental determinants [22]. Recent network analyses further support this interpretation by showing that dietary, lifestyle, and oral hygiene behaviors form interconnected behavioral communities rather than independent risk factors [28]. Consequently, the inverse association identified in the present study is compatible with the tendency for health-promoting behaviors to travel together across Brazilian population strata, rather than reflecting a direct biological effect of exercise on caries development.

The absence of a corresponding association for weekly physical activity volume is noteworthy and merits caution in interpretation. If a direct physiological pathway (e.g., exercise-induced changes in salivary flow, composition, or immune markers) contributed to this relationship, a dose-dependent pattern might be expected. Experimental evidence on this pathway remains inconsistent. Acute exercise transiently modifies several salivary characteristics, including flow rate, protein composition, and secretory immunoglobulin A secretion. However, these responses are highly dependent on exercise intensity, duration, hydration status, and individual training status, with studies reporting inconsistent or even contradictory findings [29,30,31]. Consequently, although exercise-induced changes in saliva have been proposed as a potential mechanism linking physical activity to oral health, current evidence has not consistently demonstrated a clinically meaningful effect on dental caries development. The present findings, in which only the binary engagement indicator retained significance, are therefore more compatible with a behavioral and social patterning explanation than with a dose-dependent biological mechanism, reinforcing the value of interpreting this association as a marker of population-level lifestyle clustering rather than a physiological effect of exercise dose.

The attenuation of the association between physical activity and untreated caries after adjustment for Brazilian macro-regions deserves careful interpretation. Rather than suggesting that the association is spurious, this pattern may be consistent with ecological confounding, whereby geographic units capture multiple contextual determinants that are spatially clustered and associated with both the exposure and the outcome [32,33,34]. In Brazil, macro-regions differ substantially in socioeconomic conditions, access to dental care, dietary patterns, and other structural determinants of health, all of which may influence untreated caries independently of physical activity. Consequently, part of the crude association may reflect these broader contextual characteristics rather than physical activity itself. This attenuation is therefore compatible with ecological analyses conducted at coarse geographic scales and reinforces the interpretation that physical activity should be viewed as one marker within a broader constellation of regionally patterned health determinants, rather than as an isolated protective exposure.

When oral health indicators were examined independently of physical activity, marked regional disparities remained evident, with the North region presenting a higher prevalence of untreated caries and the South exhibiting more favorable DMFT and tooth loss indicators. These geographic differences are consistent with the well-established social gradient in oral health and are more plausibly explained by structural determinants than by individual behaviors alone [27,35]. In Brazil, persistent regional inequalities in education, income, healthcare infrastructure, and access to preventive dental services continue to shape oral health outcomes despite important advances in public dental care [6]. Such contextual factors influence not only the occurrence and treatment of oral diseases but also broader patterns of health-related behaviors, including physical activity. Consequently, the regional variation observed in the present study may reflect shared upstream social determinants that simultaneously influence physical activity participation and oral health, reinforcing an interpretation based on the social patterning of health rather than a direct behavioral pathway.

Some limitations warrant consideration. As with any ecological analysis, associations observed at the population-stratum level cannot be assumed to hold at the individual level, a constraint widely recognized in population health research as the ecological fallacy. Accordingly, the present findings should be interpreted as evidence of population-level covariation rather than individual behavioral causation. In addition, the reliance on secondary data introduces potential sources of measurement bias. Physical activity was assessed through self-reported telephone interviews and may therefore be subject to recall and social desirability bias. Likewise, toothache was obtained as a self-reported outcome and may be influenced by individual pain perception, recall, health literacy, and access to dental care, meaning that the reported prevalence may not fully reflect the underlying burden of symptomatic oral disease. Furthermore, the cross-sectional nature of both surveillance systems precludes any inference regarding temporal sequence or direction of effect. Physical activity and oral health indicators were harmonized across two independently designed surveillance systems, which, despite careful alignment of stratification variables, may not capture identical population subgroups. Another important limitation is the possibility of residual socioeconomic and contextual confounding. Although the regression models were adjusted for sex, age group, and macro-region, additional contextual indicators were not incorporated because the study was designed around the harmonization of VIGITEL and SB Brasil using identical ecological strata. The post hoc inclusion of external municipal-level indicators would require additional assumptions regarding analytical comparability and could introduce further methodological heterogeneity. Consequently, the observed association between physical activity participation and untreated dental caries may be partially, substantially, or even entirely attributable to unmeasured socioeconomic and contextual confounding. Accordingly, this finding should be interpreted as hypothesis-generating rather than evidence of an independent or causal association. Future multilevel studies integrating contextual socioeconomic indicators are needed to evaluate the robustness of this association. Finally, multiple regression models were fitted to examine two prespecified physical activity indicators across five conceptually distinct oral health outcomes. Because these analyses addressed a priori hypotheses rather than data-driven screening, formal adjustment for multiple comparisons was not performed. Nevertheless, we explicitly acknowledge the issue of multiplicity arising from the ten exposure–outcome tests performed. In this context, the single nominally significant association observed for untreated dental caries may represent a chance finding and should not be interpreted as confirmatory evidence. Therefore, the main finding of the present study should be regarded as hypothesis-generating and require confirmation in future independent studies.

Notwithstanding these limitations, this study offers several strengths. To our knowledge, it is the first nationwide analysis to jointly examine physical activity and multiple oral health indicators using population-based Brazilian surveillance data, linking two independent national systems that had not previously been analyzed together. The consistency of the untreated caries association across adjustment models, together with its convergence with individual-level evidence from other populations [11,12,25], reinforces its epidemiological plausibility. From a public health perspective, these findings are consistent with the Common Risk Factor Approach and may suggest that physical activity and oral health should be considered within broader health promotion strategies targeting shared behavioral and socioeconomic determinants. Future studies employing individual-level, longitudinal designs across diverse Brazilian populations will be necessary to determine whether this population-level pattern reflects a direct behavioral pathway, shared social determinants, or both, and to consolidate physical activity as a component of integrated oral health promotion strategies.

## 5. Conclusions

Higher population-level participation in physical activity was associated with a lower prevalence of untreated dental caries, whereas no consistent associations were observed for tooth loss, dental pain, or the DMFT index. These findings are consistent with the concept that oral health and physical activity are embedded within the same network of social and behavioral determinants, as proposed by the Common Risk Factor Approach. Although causal inference is not possible from this ecological design, the observed association may reflect shared upstream determinants influencing both physical activity and oral health rather than a direct relationship between them. Future longitudinal and multilevel studies are needed to clarify the individual and contextual pathways underlying these associations.

## Figures and Tables

**Table 1:** Description of the study variables, measurement units, and data sources.

Variable	Source	Type	Unit	Description
Physical activity participation	VIGITEL	Exposure	%	Percentage of adults reporting regular physical activity.
Weekly physical activity volume	VIGITEL	Exposure	minutes/week	Mean weekly volume of physical activity among participants.
Untreated dental caries	SB Brasil	Outcome	%	Prevalence of individuals presenting at least one untreated carious lesion.
Toothache	SB Brasil	Outcome	%	Prevalence of self-reported toothache.
Tooth pain intensity	SB Brasil	Outcome	Score	Mean self-reported tooth pain intensity.
DMFT index	SB Brasil	Outcome	Mean score	Mean DMFT index
Missing teeth	SB Brasil	Outcome	Mean teeth	Mean number of missing teeth.
Sex	Both	Covariate	Male/Female	Biological sex used for data stratification.
Age group	Both	Covariate	Categories	Age groups used for data aggregation.
Brazilian macro-region	Both	Covariate	Five regions	North, Northeast, Central-West, Southeast and South.

DMFT—Decayed, Missing, and Filled Teeth; VIGITEL—Surveillance System for Risk and Protective Factors for Chronic Diseases by Telephone Survey; SB Brasil—Brazilian National Oral Health Survey.

## Data Availability

The datasets analyzed in this study are publicly available from the Brazilian Ministry of Health. VIGITEL data are available at https://svs.aids.gov.br/download/Vigitel/, (accessed on 4 May 2026) and SB Brasil 2023 data are available at https://www.gov.br/saude/pt-br/composicao/saps/brasil-sorridente/sb-brasil (accessed on 4 May 2026). No new datasets were generated during the current study.

## Supplementary Materials

The following supporting information can be downloaded at https://www.mdpi.com/article/10.3390/dj14080514/s1.
