# Supplementary material for: Physical Activity and Untreated Dental Caries in Brazil: Implications for Population-Based Caries Risk Assessment
Source: Dent J (Basel). 2026 Aug 12;14(8):514. doi: 10.3390/dj14080514 (PMC13510501; doi:10.3390/dj14080514)
Supplement: Supplementary file 1 [file dentistry-14-00514-s001.zip › Supplementary File S2.pdf]

Supplementary File S2

Supplementary File S2 presents the geographic distribution of physical activity and oral health indicators across Brazilian capitals (2022–2023), including physical activity prevalence, weekly physical activity volume, untreated dental caries, toothache prevalence, toothache intensity, DMFT index, and tooth loss.

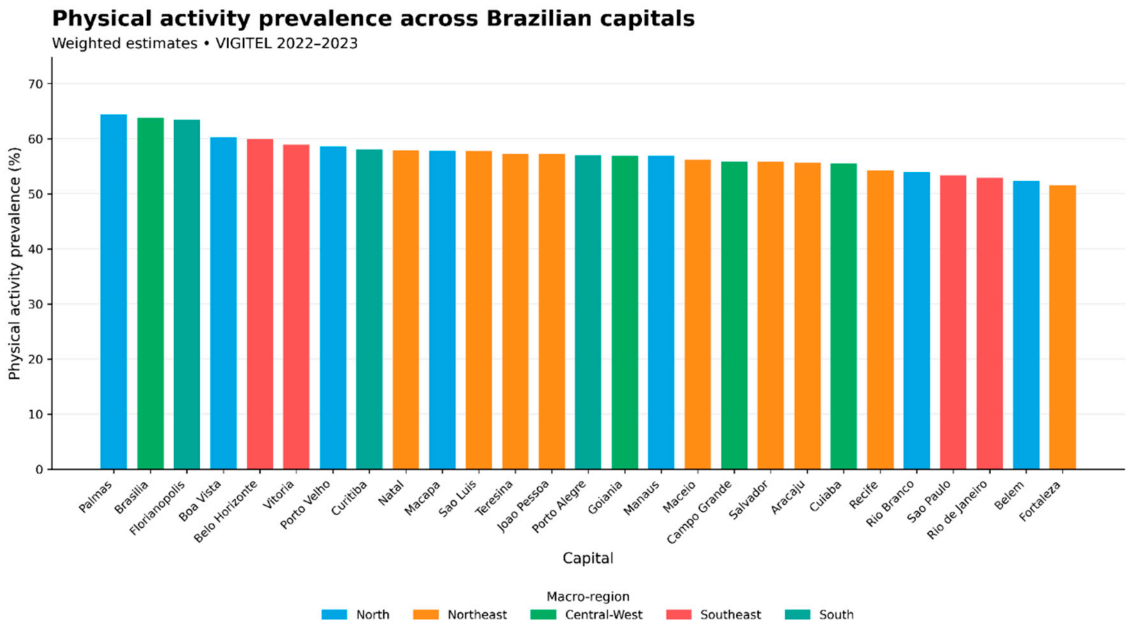

Supplementary Figure S1. Distribution of physical activity prevalence across Brazilian capitals (2022–2023). Values represent weighted estimates by capital, with colors indicating macro-region.

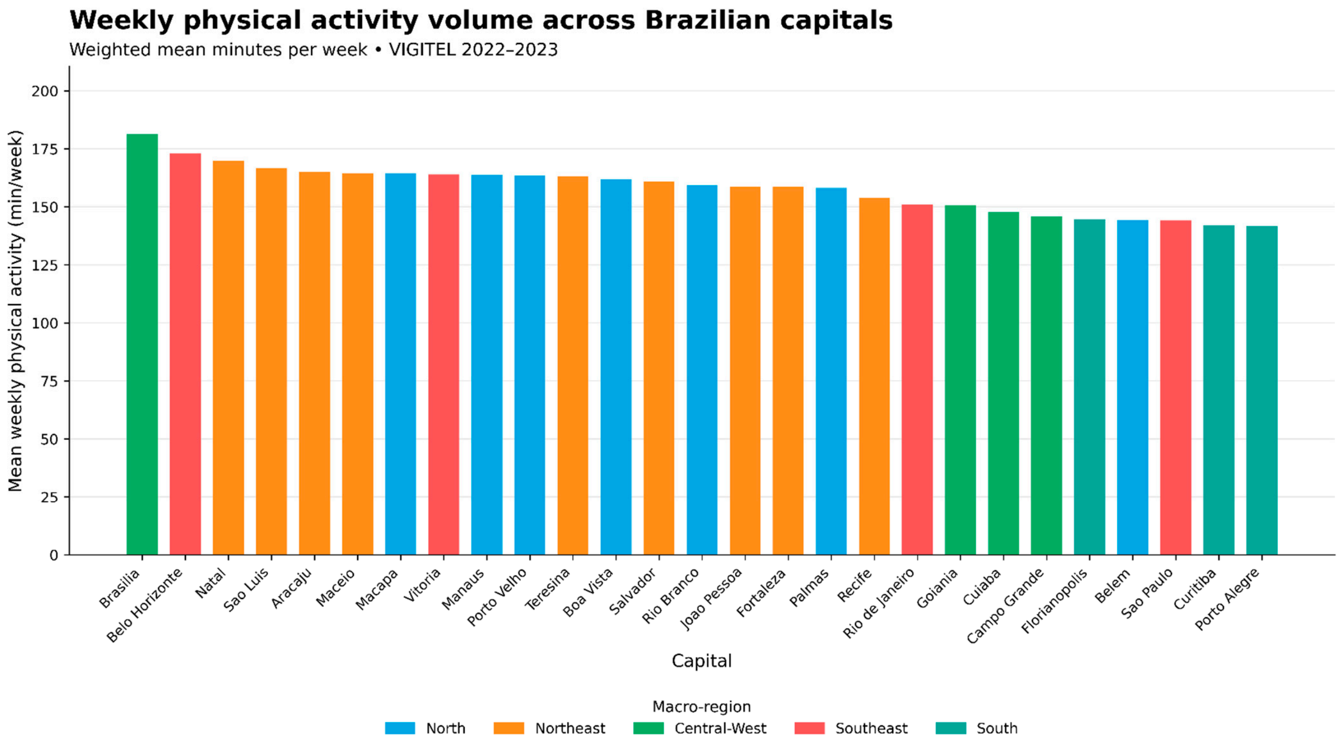

Supplementary Figure S2. Distribution of weekly physical activity volume across Brazilian capitals (2022–2023). Values represent weighted mean minutes per week by capital, with colors indicating macro-region.

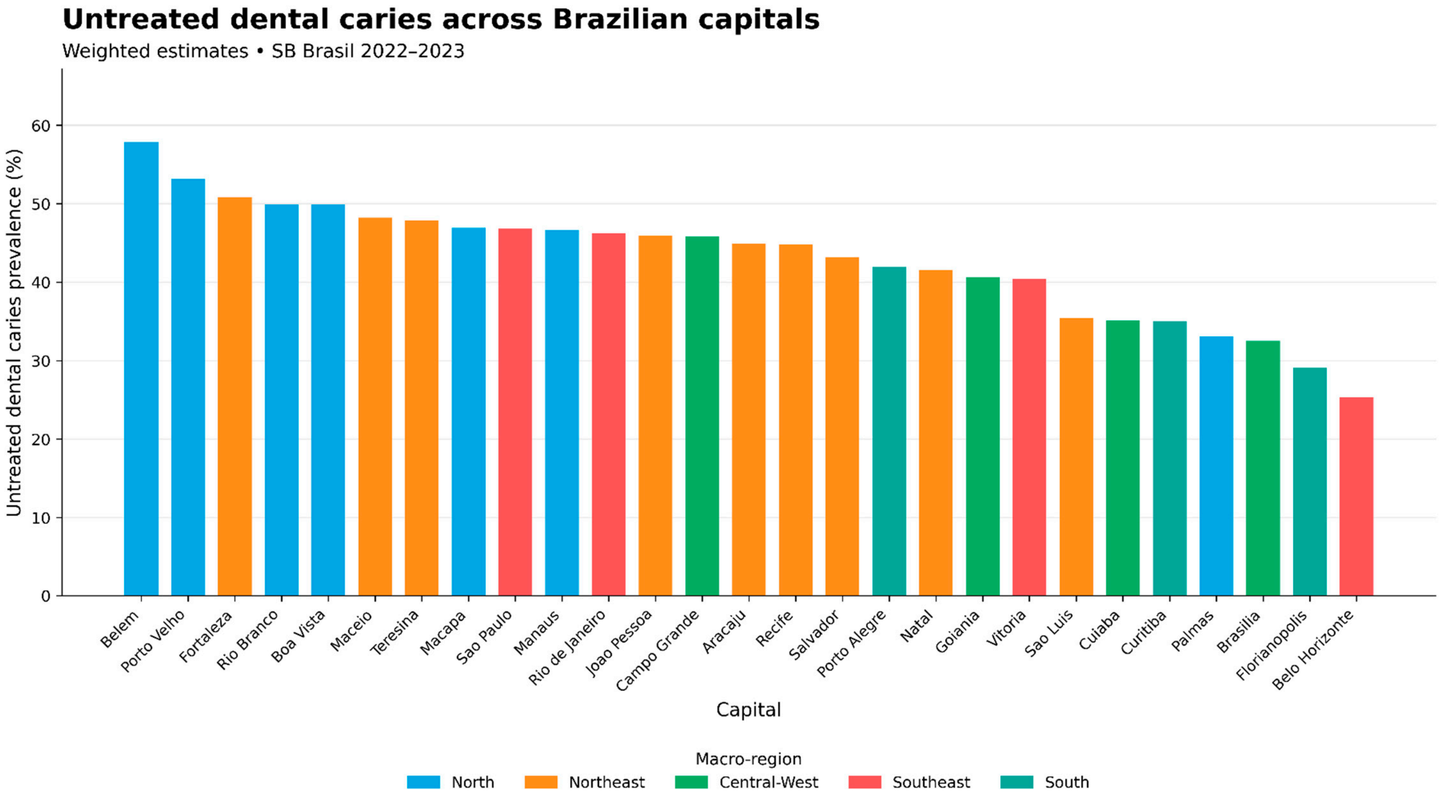

Supplementary Figure S3. Distribution of untreated dental caries prevalence across Brazilian capitals (2022–2023). Values represent weighted estimates by capital, with colors indicating macro-region.

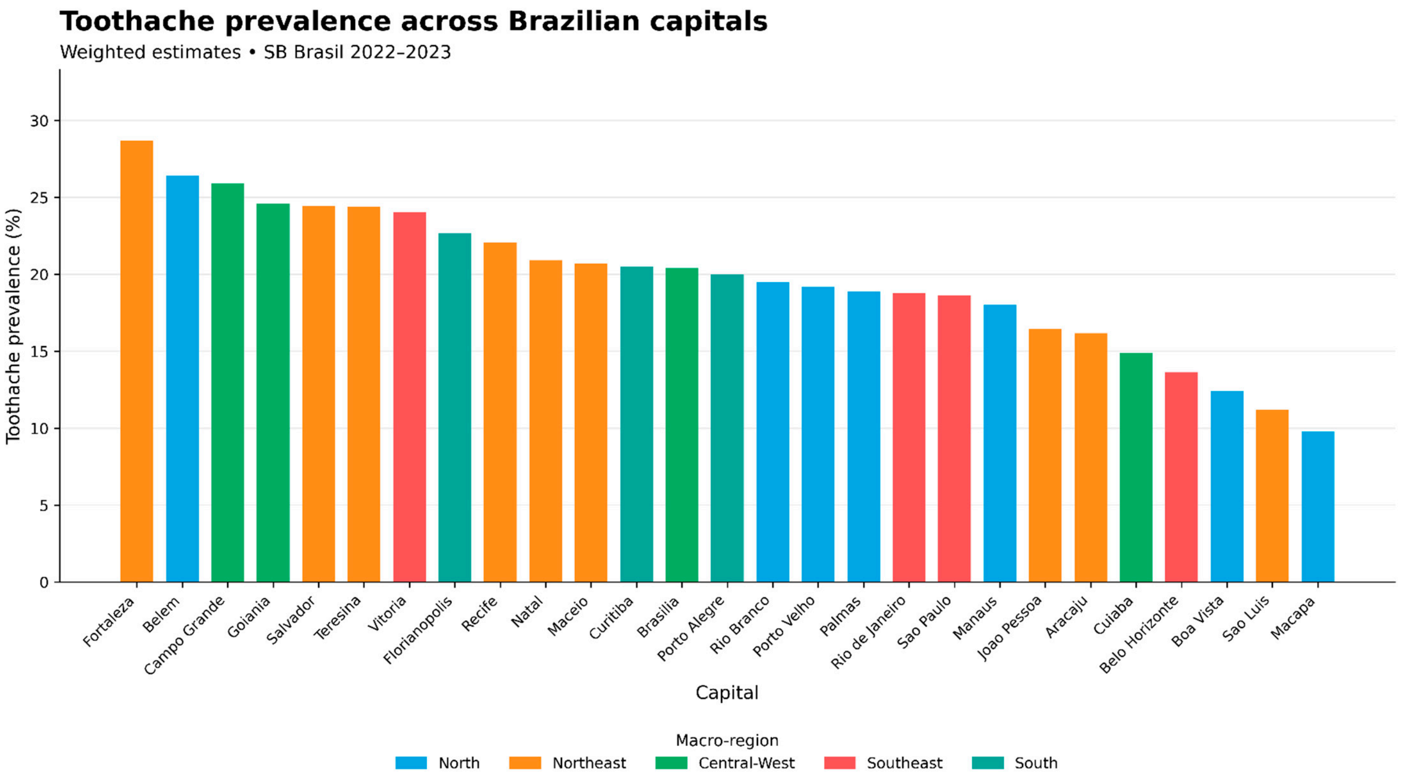

Supplementary Figure S4. Distribution of toothache prevalence across Brazilian capitals (2022–2023). Values represent weighted estimates by capital, with colors indicating macro-region.

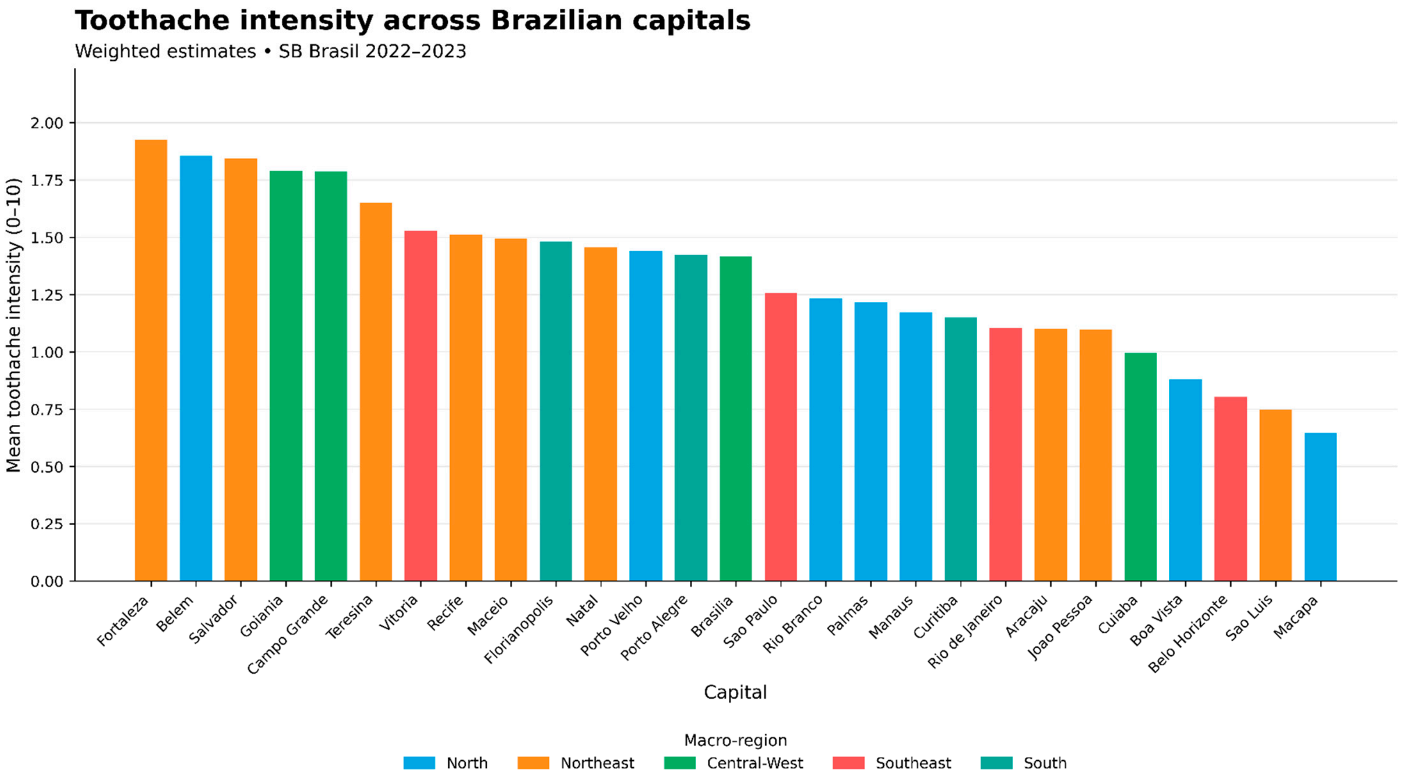

Supplementary Figure S5. Distribution of mean toothache intensity across Brazilian capitals (2022–2023). Values represent weighted estimates by capital, with colors indicating macro-region.

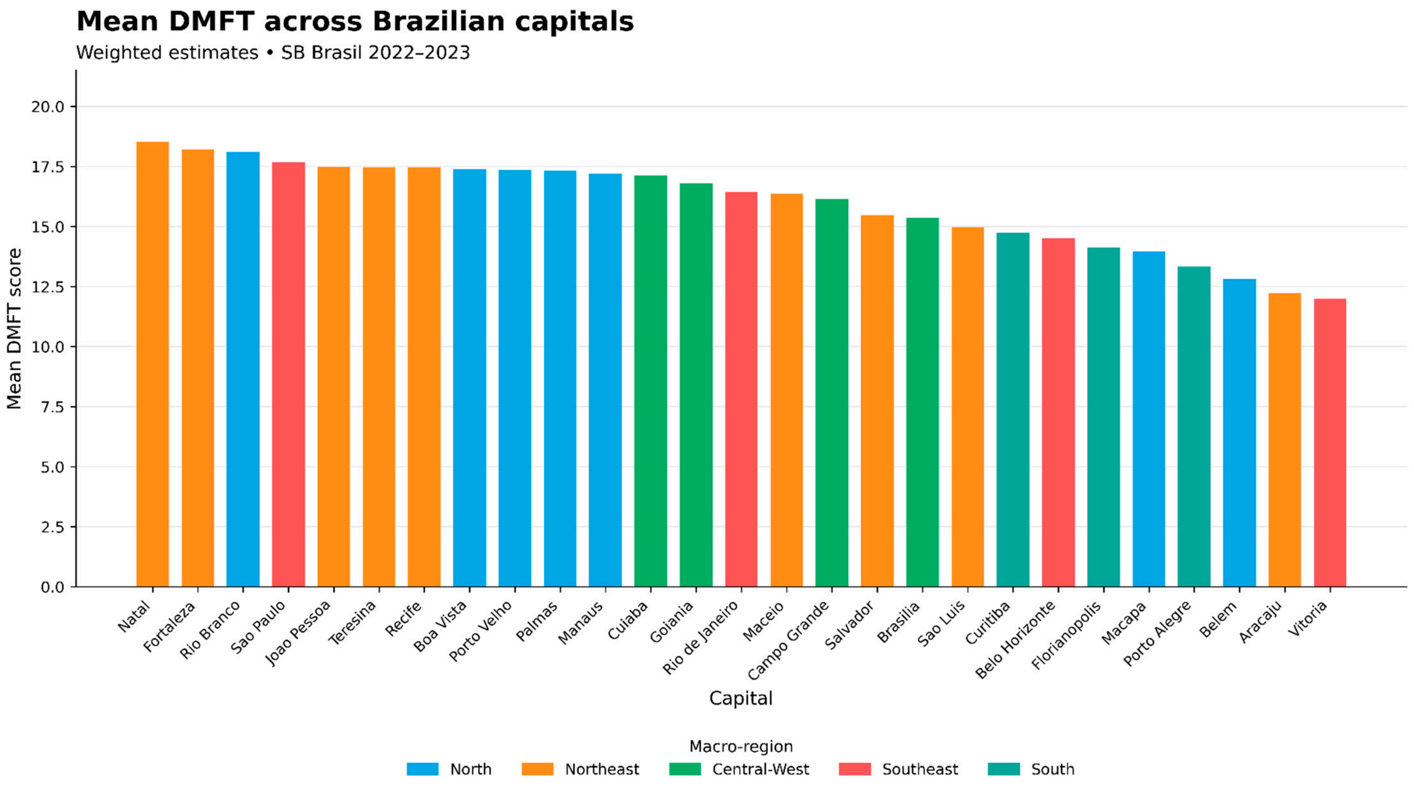

Supplementary Figure S6. Distribution of mean Decayed, Missing, and Filled Teeth index (DMFT) across Brazilian capitals (2022–2023). Values represent weighted estimates by capital, with colors indicating macro-region.

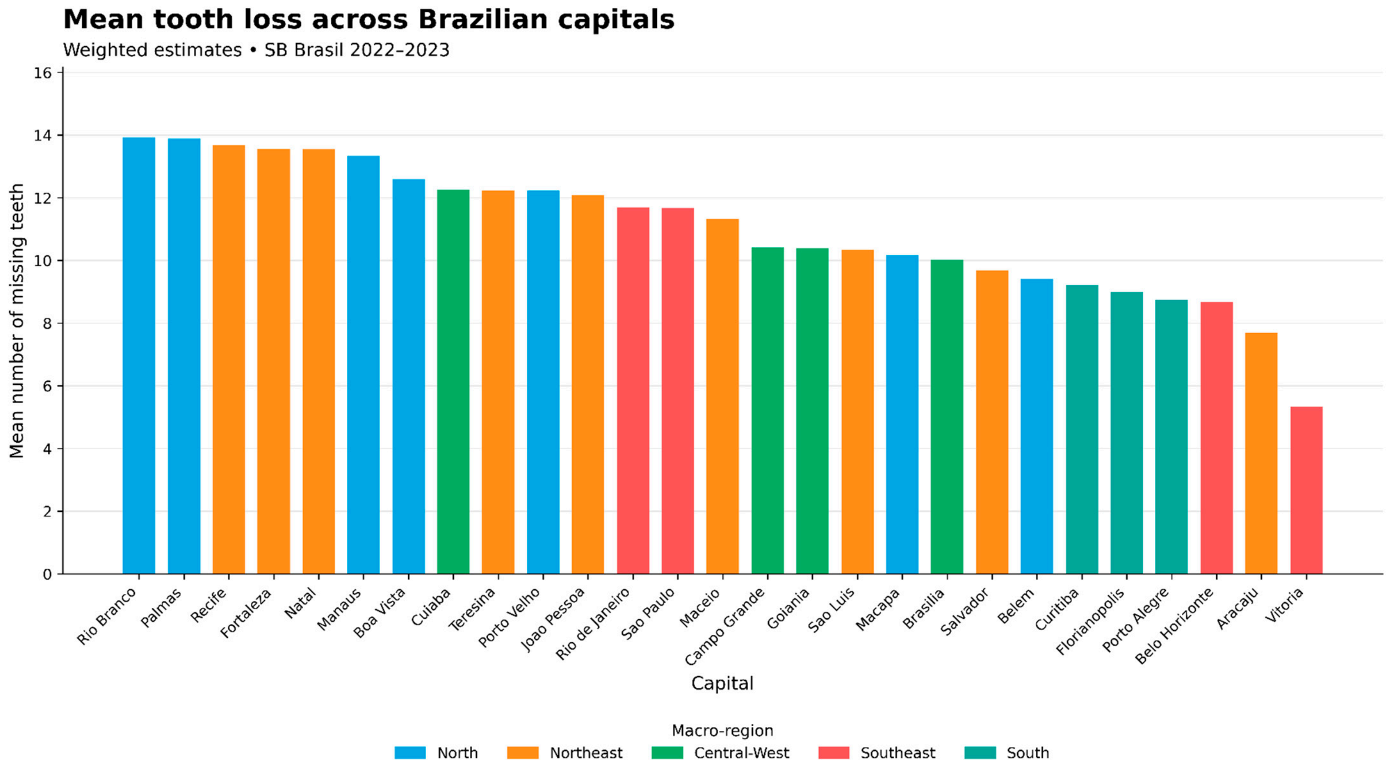

Supplementary Figure S7. Distribution of mean tooth loss across Brazilian capitals (2022–2023). Values represent weighted estimates by capital, with colors indicating macro-region.
