# Supplementary material for: Physical Activity and Untreated Dental Caries in Brazil: Implications for Population-Based Caries Risk Assessment
Source: Dent J (Basel). 2026 Aug 12;14(8):514. doi: 10.3390/dj14080514 (PMC13510501; doi:10.3390/dj14080514)
Supplement: Supplementary file 1 [file dentistry-14-00514-s001.zip › Supplementary File S3.pdf]

Supplementary File S3

Supplementary File S3 provides the complete weighted multivariable linear regression results for all oral health outcomes, including regression coefficients (β), standard errors, test statistics, p-values, and model fit indices (R and R2).

Supplementary Table S1. Model fit measures, predictors, and estimates for untreated caries.

Model		R		R^2
Untreated caries		0.829		0.687
**Predictor**	**Estimates (β)**	**Standard-error**	**t**	**p**
Intercept	0.500	0.123	4.066	0.000*
Physical activity prevalence	-0.346	0.151	-2.288	0.023*
Mean weekly physical activity volume	0.001	0.000	1.609	0.109
**Sexo:**				
Male – Female	0.081	0.023	3.511	0.001*
**Age:**				
30-39 – 18-29 yrs	0.112	0.032	3.504	0.001*
40-49 – 18-29 yrs	0.079	0.033	2.400	0.017*
60+ – 18-29 yrs	-0.166	0.042	-3.930	0.000*
**Macro-region:**				
Northeast – Central-west	0.032	0.025	1.264	0.208
North – Central-west	0.067	0.025	2.625	0.009*
Southeast – Central-west	0.053	0.028	1.892	0.060
South – Central-west	-0.035	0.027	-1.292	0.198

*indicates significant relationship.

Supplementary Table S2. Model fit measures, predictors, and estimates for toothache.

Model	R	R^2
Toothache	0.700	0.490

Predictor	Estimates (β)	Standard-error	t	p
Intercept	0.296	0.100	2.961	0.003*
Physical activity prevalence	0.042	0.111	0.377	0.706
Mean weekly physical activity volume	0.000	0.000	-0.542	0.588
**Sexo:**				
Male – Female	-0.020	0.017	-1.189	0.236
**Age:**				
30-39 – 18-29 yrs	0.003	0.026	0.118	0.906
40-49 – 18-29 yrs	-0.011	0.025	-0.425	0.671
60+ – 18-29 yrs	-0.140	0.032	-4.339	0.000*
**Macro-region:**				
Northeast – Central-west	-0.006	0.022	-0.286	0.776
North – Central-west	-0.047	0.023	-2.057	0.041*
Southeast – Central-west	-0.012	0.023	-0.519	0.604
South – Central-west	-0.001	0.022	-0.027	0.979

*indicates significant relationship.

Supplementary Table S3. Model fit measures, predictors, and estimates for toothache intensity.

Model	R	R^2
Toothache intensity	0.700	0.490

Predictor	Estimates (β)	Standard-error	t	p
Intercept	1.916	0.749	2.556	0.011*
Physical activity prevalence	0.254	0.845	0.301	0.764
Mean weekly physical activity volume	-0.001	0.003	-0.249	0.804
**Sexo:**				
Male – Female	-0.176	0.131	-1.342	0.181
**Age:**				
30-39 – 18-29 yrs	0.127	0.190	0.666	0.506
40-49 – 18-29 yrs	0.029	0.185	0.159	0.874
60+ – 18-29 yrs	-0.938	0.240	-3.911	0.000*
**Macro-region:**				
Northeast – Central-west	-0.052	0.167	-0.312	0.756
North – Central-west	-0.359	0.171	-2.093	0.038*
Southeast – Central-west	-0.209	0.173	-1.206	0.229
South – Central-west	-0.120	0.169	-0.708	0.480

*indicates significant relationship.

Supplementary Table S4. Model fit measures. predictors. and estimates for DMFT.

Model		R		R^2
DMFT		0.968		0.936
**Predictor**	**Estimates (β)**	**Standard-error**	**t**	**p**
Intercept	5.061	2.539	1.993	0.048
Physical activity prevalence	0.980	2.813	0.348	0.728
Mean weekly physical activity volume	-0.002	0.011	-0.160	0.873
**Sexo:**				
Male – Female	-0.727	0.454	-1.604	0.111
**Age:**				
30-39 – 18-29 yrs	4.519	0.472	9.573	0.000*
40-49 – 18-29 yrs	6.925	0.596	11.626	0.000*
60+ – 18-29 yrs	18.972	0.740	25.625	0.000*
**Macro-region:**				
Northeast – Central-west	-0.159	0.603	-0.264	0.792
North – Central-west	-0.153	0.601	-0.254	0.800
Southeast – Central-west	-0.965	0.676	-1.428	0.155
South – Central-west	-2.870	0.619	-4.640	0.000*

DMFT - Decayed. Missing. and Filled Teeth index; *indicates significant relationship.

Supplementary Table S5. Model fit measures. predictors. and estimates for tooth loss.

Model	R	R^2
Tooth loss	0.971	0.943

Predictor	Estimates (β)	Standard-error	t	p
Intercept	1.692	2.642	0.641	0.523
Physical activity prevalence	1.443	2.975	0.485	0.628
Mean weekly physical activity volume	-0.009	0.010	-0.853	0.395
**Sexo:**				
Male – Female	-0.677	0.522	-1.299	0.196
**Age:**				
30-39 – 18-29 yrs	1.602	0.495	3.236	0.001*
40-49 – 18-29 yrs	3.295	0.599	5.501	0.000*
60+ – 18-29 yrs	19.100	0.802	23.804	0.000*
**Macro-region:**				
Northeast – Central-west	0.636	0.800	0.795	0.428
North – Central-west	1.464	0.757	1.935	0.055
Southeast – Central-west	-1.021	0.850	-1.201	0.231
South – Central-west	-2.529	0.878	-2.879	0.004*

DMFT - Decayed. Missing. and Filled Teeth index; *indicates significant relationship.
